# Compatibilized Immiscible Polymer Blends for Gas Separations

**DOI:** 10.3390/ma9080643

**Published:** 2016-07-30

**Authors:** Nimanka Panapitiya, Sumudu Wijenayake, Do Nguyen, Chamaal Karunaweera, Yu Huang, Kenneth Balkus, Inga Musselman, John Ferraris

**Affiliations:** Department of Chemistry and Biochemistry, The University of Texas at Dallas, 800 W. Campbell Rd, Richardson, TX 75080-3021, USA; nimanka.panapitiya@utdallas.edu (N.P.); snw081000@utdallas.edu (S.W.); Do.Nguyen@utdallas.edu (D.N.); cxk140730@utdallas.edu (C.K.); yxh091220@utdallas.edu (Y.H.); balkus@utdallas.edu (K.B.J.); imusselm@utdallas.edu (I.M.)

**Keywords:** immiscible polymer blends, compatibilizers, gas separation membranes

## Abstract

Membrane-based gas separation has attracted a great deal of attention recently due to the requirement for high purity gasses in industrial applications like fuel cells, and because of environment concerns, such as global warming. The current methods of cryogenic distillation and pressure swing adsorption are energy intensive and costly. Therefore, polymer membranes have emerged as a less energy intensive and cost effective candidate to separate gas mixtures. However, the use of polymeric membranes has a drawback known as the permeability-selectivity tradeoff. Many approaches have been used to overcome this limitation including the use of polymer blends. Polymer blending technology synergistically combines the favorable properties of different polymers like high gas permeability and high selectivity, which are difficult to attain with a single polymer. During polymer mixing, polymers tend to uncontrollably phase separate due to unfavorable thermodynamics, which limits the number of completely miscible polymer combinations for gas separations. Therefore, compatibilizers are used to control the phase separation and to obtain stable membrane morphologies, while improving the mechanical properties. In this review, we focus on immiscible polymer blends and the use of compatibilizers for gas separation applications.

## 1. Introduction

**Figure 1:**
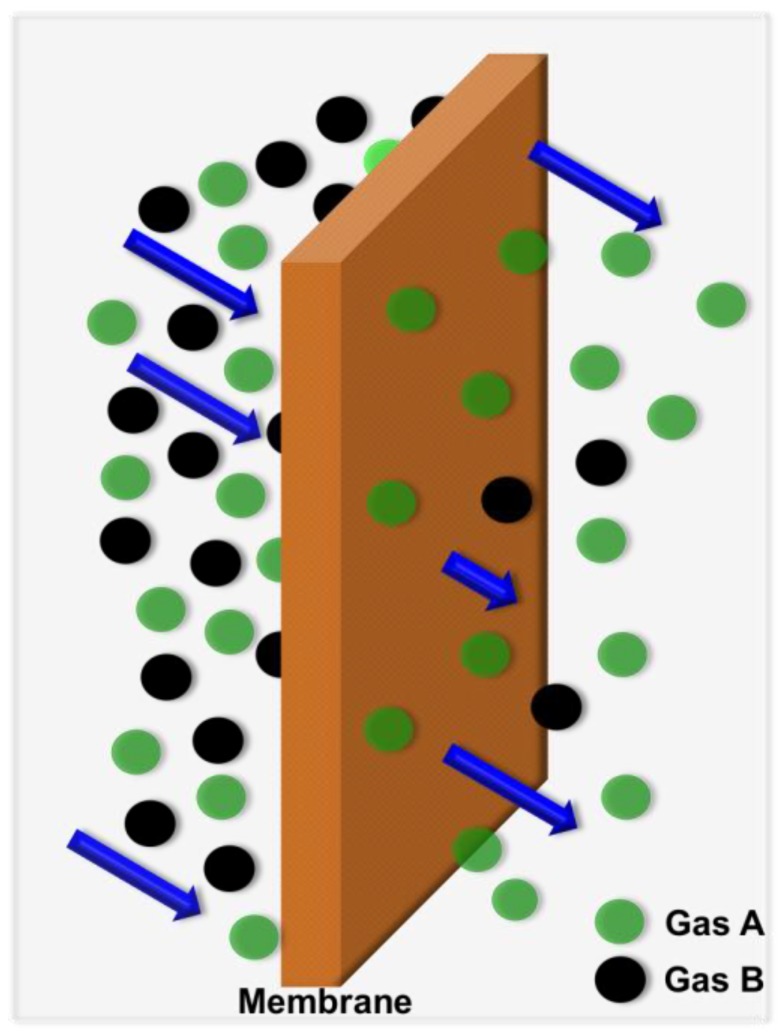
Gas transport through polymeric membranes.

The global oil and gas separation market is expected to be worth ~11 billion USD by 2020 [1]. Conventional methods of gas separation include pressure swing adsorption [2,3] and fractional/cryogenic distillation [4]. The main disadvantages of these methods are their high costs and high-energy consumptions. Membrane technology [5,6,7,8,9,10,11,12,13,14] has emerged as a promising method of gas separation because of its low energy consumption, the possibility of continuous operation, which dramatically lowers the investment cost, its ease of operation, and cost effectiveness [15]. Membrane technology is currently being used commercially in natural gas purification, CO_2_ capture, hydrogen recovery, and oxygen and nitrogen enrichment [16]. For efficient gas separation, membranes should be highly selective, highly permeable [17], and durable. The transport of gases across a dense polymeric membrane can be explained using the solution-diffusion model [18,19]. According to this model, gas molecules enter the polymer membrane from the upstream side, dissolve in the membrane matrix, are transported across the membrane through a partial pressure gradient, and then desorb from the downstream side (Figure 1).

The permeation of gas molecules through a membrane depends on the diffusivity (*D*) and solubility (*S*) of the different gases (Equation (1)). Diffusivity describes the mobility of individual gas molecules passing through the free volume of a membrane and depends on the kinetic diameter of the gas, with smaller molecules diffusing faster. Solubility is a measure of the quantity of gas molecules dissolved in a membrane material and is directly related to the condensability of the gas.

The permeability (P) of a gas through a membrane can, therefore, be defined as:

P = D × S(1)

The selectivity (α), or separation factor, measures the ability of a membrane to separate two different gas molecules and is equal to the ratio of their respective gas permeabilities and is determined by the balance between the solubility selectivity (S_A_/S_B_) and the diffusivity selectivity (D_A_/D_B_) (Equation (2)).

α_A/B_ = P_A_/P_B_ = (D_A_/D_B_) × (S_A_/S_B_) (2)

**Figure 2:**
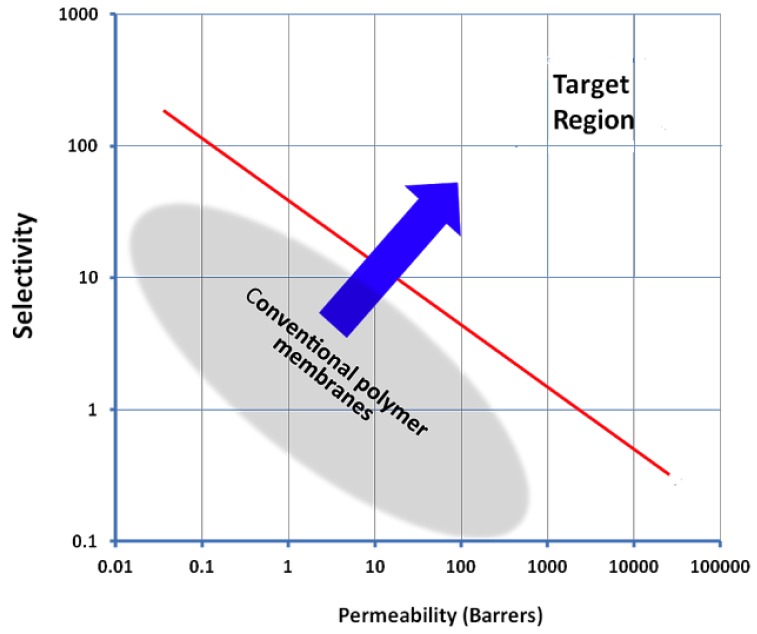
Robeson upper bound for gas separations.

Polymeric membranes for gas separation display a trade-off between gas permeability and selectivity as demonstrated by Robeson with his upper bound curve, first in 1991 [20] and later updated in 2008 [21]. One of the motivations of current membrane research is to develop membranes that surpass the upper bound (Figure 2) with separation performance in the commercially desirable region.

Although many efforts have been made to surpass the upper bound, including the synthesis of new polymers [22,23,24,25], cross-linking of polymers [26,27], fabrication of inorganic-organic composite materials (mixed-matrix membranes) [8,28,29,30], cross-linking of mixed matrix membranes [31,32], carbon molecular sieve membranes (CMSM) [33,34,35,36], and use of polymer blends [37,38,39,40,41], only a very few systems have exceeded this two-decade old limit.

Blending is a simple and effective technique to synergistically combine favorable properties of different polymers such as high gas permeability and high gas selectivity [37,38]. This approach is economically advantageous over the synthesis of novel materials, which can be time-consuming and costly. It is no surprise, then, that polymer blends have been used for many applications including photovoltaics [42], light emitting diodes [43], food packaging [44], fuel cells [45], energy storage devices [46], and gas separation membranes as mentioned above [37,38,39,40,41]. In a recent review article on polymer blends, Mannan et al. discuss the enhancement in both gas permeability and selectivity for miscible polymer blends, such as polyimides with polybenzimidazole (PBI), polyether ether ketone (PEEK), polyethersulfone (PES), and sulfonated PEEK [13]. It has been observed that blending also improves the mechanical properties of the polymers. The miscibility of polymer systems depends on both thermodynamic and kinetic factors. A completely miscible blend exhibits a uniform microstructure throughout the membrane and generally affords gas separation properties intermediate between the component polymers. Knowing the fractions of the polymers in the blend and the permeabilities of the individual polymers, Equation (3) can be used to calculate the permeability (*P_b_*) values of miscible polymer blend membranes. Here, *Ø_1_*, *Ø_2_* and *P_1_*, *P_2_* are volume fractions and permeabilities, respectively, of the components of the miscible binary polymer blend.

(3) $$\ln P_{b}={Ø}_{1}\ln P_{1}+{Ø}_{2}\ln P_{2}$$

However, the number of polymer pairs that form miscible blends is limited due to the unfavorable entropy of mixing [47]. The number of immiscible blends, however, is essentially limitless, but care must be taken to avoid uncontrolled phase separation. In immiscible (phase separated) polymer blend membranes, the gas separation properties depend on the membrane morphology [48]. For example, phase separated polymer blends can exhibit co-continuous, matrix-droplet, fiber, and lamellar morphologies [49]. For gas separation applications, matrix-droplet morphology is advantageous due to the high interfacial surface area it affords.

The Maxwell model (Equation (4)) can be used to predict gas permeabilities of immiscible polymer blends with matrix-droplet morphology. Here, *P_d_* and *P_c_* are permeabilities of the dispersed and continuous phases, respectively, while *Ø_d_* and *Ø_c_* are the volume fractions of dispersed and continuous phases, respectively [48].

(4) $$P_{b}=P_{c}\left[\frac{P_{d}+2P_{c}-2{Ø}_{d}\left(P_{c}-P_{d}\right)}{P_{d}+2P_{c}-{Ø}_{d}\left(P_{c}-P_{d}\right)}\right]$$

The use of immiscible polymer blends is limited by their uncontrolled phase separation, which results in poor mechanical properties [50,51] and diverse microstructures [52]. Therefore, to improve polymer miscibility and to obtain uniform microstructures, compatibilizers, including co-polymers [53,54,55], nanoparticles [56,57], metal-organic frameworks (MOFs) [58], and commercially available small molecules [59] have been employed. The use of these compatibilizers and the potential applications of the blends in gas separations are reviewed here.

## 2. Miscible Polymer Blends for Gas Separations

**Figure 3:**
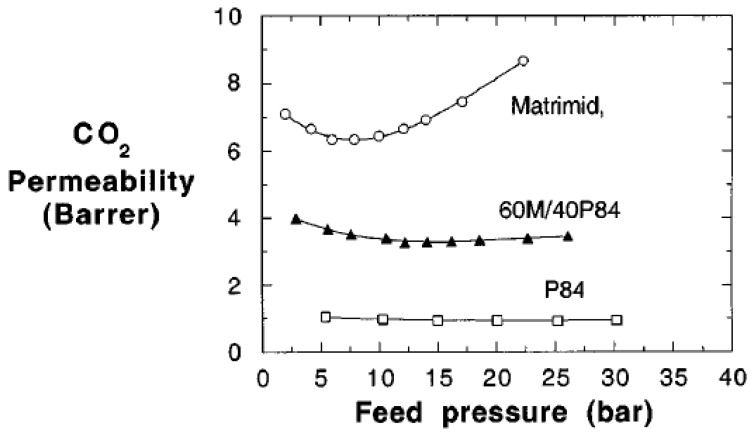
Variation of CO_2_ permeability with feed pressure for 60 wt % Matrimid^®^/40 wt % P84^®^ miscible blend membrane [65]. (Reprinted with permission from Bos, A.; Pünt, I.; Strathmann, H.; Wessling, M. Suppression of gas separation membrane plasticization by homogeneous polymer blending. *AIChE J.* **2001**, *47*, 1088–1093. Copyright 2001 AlChE.)

Earliest reports on polymer blends for gas separation utilized miscible polymers. In 1982, Morel et al. studied CO_2_ sorption and transport through the miscible polymer blend comprising polyphenyleneoxide (PPO)/polystyrene (PS) [60]. CO_2_ permeability decreased for the blends due to strong interactions between the two polymers [60]. Maeda et al. were able to improve He/CH_4_ and CO_2_/CH_4_ separation factors using PPO/PS blends in contrast to the pure polymers [61]. Polystyrene/polycarbonate based membranes were tested for O_2_/N_2_, CO_2_/CH_4_ and He/CH_4_ separations by Muruganandam et al., in 1987. They were, however, unable to achieve improvements for the above gas pair selectivities [62]. Focusing on the improvements in mechanical properties of membranes and O_2_/N_2_ separation factor, Lokaj and co-workers carried out a study on poly(N-(3-dimethylaminophenyl)maleimide)/poly(2,6-dimethyl-1,4-phenylene oxide) blend membranes. They obtained mechanically and thermally stable miscible polymer blend membranes having improved O_2_/N_2_ separation performances [63]. Kapantaidakis et al. blended polysulfone (PSF) with Matrimid^®^ polyimide (PI) [64]. Pure PI membranes were susceptible to plasticization, which limited their use at higher CO_2_ pressures. However, upon blending with PSF, the plasticization pressure increased from 15 (for pure PI) to ~30 atm (for PI/PSF 50 wt %: 50 wt % blend) [64]. Bos et al. reported a plasticization pressure improvement for a Matrimid^®^/P84^®^ (60/40) miscible blend [65]. The CO_2_ plasticization pressure for the blend membrane (15 bar) fall in between those for the pure polymers (Matrimid^®^ 9 bar, P84^®^ 22 bar). Figure 3 displays the variation of CO_2_ permeability of pure P84^®^, pure Matrimid^®^ and 60 wt % Matrimid^®^/40 wt % P84^®^ blend membranes. Moreover, the CO_2_ permeabilities and CO_2_/CH_4_ selectivities for the blend fall in between the values recorded for pure Matrimid^®^ and P84^®^ [65].

Several researchers have studied flat membranes utilizing polymer blends to enhance gas pair separations [39,61,66,67]. Frequently, polyimides were blended with another polymer having high selectivity for a particular gas pair. Polybenzimidazole (PBI) is a polymer with a high separation factor for H_2_ over other gases. However, the permeability is quiet low for PBI membranes. As a solution for this, Hosseini et al. [37], used miscible polymer blends of PBI/Matrimid^®^ to prepare H_2_ and CO_2_ selective membranes. Martimid^®^ has a H_2_ permeability of 27.16 Barrer. DSC analysis of membranes having different compositions of PBI/Matrimid^®^ showed single glass transition temperatures (T_g_), which indicated miscibility of the two polymers. The T_g_ of a miscible polymer blend can be predicted using the Fox equation (Equation (5)), where W_1_, W_2_ and T_g,1_, T_g,2_ are weight fractions and glass transition temperatures of the individual polymers, respectively [68].

(5) $$\frac{1}{T_{g}}=\frac{W_{1}}{T_{g,1}}+\frac{W_{2}}{T_{g,2}}$$

Predictions of T_g_ using the Fox equation were close to the experimental values.

Strong hydrogen bonding between the H atoms of N–H groups in PBI and O atoms of C=O groups in Matrimid^®^ were believed to be the reason for complete miscibility of the polymers [37]. For the membrane having 25/75 wt % of Matrimid^®^/PBI, H_2_ permeability was 5.47 Barrer with a H_2_/N_2_ selectivity of 260.47. Following their study on PBI/Matrimid^®^ miscible polymer blends, Hosseini et al. fabricated carbon molecular sieve membranes based on PBI/Matrimid^®^, PBI/Torlon^®^ and PBI/P84^®^ miscible blends [38]. Matrimid^®^ membranes pyrolyzed at different temperatures showed improved gas separation properties. To further improve the properties, they used chemical cross-linking of the precursor membranes using *p*-xylene diamine. Results showed gas separation properties which surpass the Robeson upper bound for gas pairs including, H_2_/CO_2_, H_2_/N_2_, CO_2_/CH_4_, N_2_/CH_4_ and H_2_/CH_4_. Table 1 shows gas permeability and ideal selectivities for PBI/Matrimid^®^ (50/50 wt %) pyrolyzed membranes (800 °C), with and without chemical cross-linking [38].

In another study, Khan et al. [39] studied the gas separation properties of polymer blend membranes comprised of Matrimid^®^ 9725 and sulfonated aromatic polyether ether ketone (SPEEK), which formed completely miscible polymer blends over the entire composition range they tested as characterized by DSC studies. Both pure and mixed gas experiments were carried out for several gases including CO_2_, CH_4_ and N_2_. Permeabilities for blend membranes were in between the values for pure polymers. With increasing SPEEK amounts, permeabilities of all three gases increased while ideal selectivities for CO_2_/CH_4_ decreased. Surprisingly, CO_2_/N_2_ ideal selectivity remained the same at ~35. In mixed-gas experiments, due to plasticization, CO_2_ permeability increased with pressure. At the same time, membranes having higher SPEEK content showed improved plasticization resistance going up to 40 bar [39]. 

Mixed matrix membranes (MMMs) have been extensively used to improve gas transport through polymeric membranes [66]. Additives, such as zeolites, MOFs, and ZIFs, were tested by combining with different polymers. In 2008, Ismail et al. [67] fabricated MMMs using polyethersulfone (PES)/Matrimid^®^ 5218 blends with zeolite 4A as the inorganic additive. Zeolite loading was changed between 10 and 50 wt %. By annealing membranes at 280 °C (above the T_g_ of the polymer blend), the membranes obtained a separation factor of approximately five times that of pure blend membrane for O_2_/N_2_ gas pair. Although the selectivities improved, permeabilities for both gases were lowered for membranes annealed at higher temperatures due to improved adhesion between the polymer chains and zeolite particles [67]. 

Hollow-fiber membranes are another type of membrane that can be used for gas separation and generally display good mechanical properties. Miscible polymer blends can be used to prepare hollow fibers. Following their work on flat membranes, Hosseini et al. [69] studied hollow-fiber membranes comprising PBI/Matrimid^®^ polymer blends. Hollow fiber membranes were made by coating a layer of silicon rubber and cross-linking the polymers with *p*-xylenediamine. Cross-linked hollow fiber membranes displayed H_2_/CO_2_ selectivity of 14.49 in comparison to 11.11 for uncross-linked membrane. However, crosslinking showed the opposite effect on CO_2_/CH_4_ selectivity. Nevertheless, the blend membranes were resistant to CO_2_ induced plasticization, which is a major drawback in pure Matrimid^®^ based membranes [69].

The use of miscible polymer blends for gas separations has been attractive and thoroughly studied. However, obtaining completely miscible polymers pairs is rare due to the unfavorable thermodynamics for polymer mixing. To overcome this barrier preferential interactions such as H bonding between polymers should be present. In order to form such H bonds specific functional groups need to be incorporated in polymers, which is synthetically challenging. Additionally, on many occasions as a result of complete miscibility, a homogeneous microstructure is obtained and the gas permeability properties lie in-between the parent polymers. 

The goal is to blend immiscible polymers whose properties can combine synergistically while maintaining control over the microstructure. We discuss various modes of compatibilization in the next section. 

## 3. Immiscible Polymer Blends in Gas Separation Membranes 

### Thermodynamics

In order for two polymers to be miscible with each other, the Gibbs free energy, *G*, of the blend, *G_12_*, should be lower than the sum of the *G* values of each polymer (Equation (6)).

(6) $$\Delta G_{m}=G_{12}-\left(G_{1}+G_{2}\right)<0$$

The change in free energies, $\Delta G_{m}$, depends on the enthalpy, $\Delta H_{m}$, and entropy, $\Delta S_{m}$, changes during mixing (Equation (7)).

(7) $$\Delta G_{m}=\Delta H_{m}-T\Delta S_{m}$$

The $\Delta H_{m}$ term represents the extent of interaction between polymers upon mixing, and $\Delta S_{m}$ represents the number of different ways available of arranging the polymers. It is important to note that usually the $\Delta S_{m}$ term is a positive quantity during mixing for small molecules since mixing increases disorder in a system. Therefore, at high temperatures, small molecules tend to dissolve readily since $-T\Delta S_{m}$ makes the $\Delta G_{m}$ more negative. However, this is not the case for polymers due to their large size. Polymers have disorder to begin with, and during mixing with another polymer, they have to obtain a certain ordered arrangement instead. Therefore, to compensate, the enthalpic term has to contribute by forming favorable interactions between polymers, such as hydrogen bonding, which drives the $\Delta S_{m}$ term negative. This phenomenon is further elaborated using the Flory-Huggins (FH) model in Equation (8),

(8) $$\frac{\Delta G_{m}}{RT}=\left(\frac{V}{V_{R}}\right)\left[\frac{\varphi_{1}}{r_{1}}ln\varphi_{1}+\frac{\varphi_{2}}{r_{2}}ln\varphi_{2}+\varphi_{1}\varphi_{2}\chi_{12}\right]$$

where *R* is the gas constant; *T* is the absolute temperature; *V* is the total molar volume; *r_i_*, the number of segments per chain volume or the degree of polymerization of polymer *i* relative to the reference volume *V_R_*; $\varphi_{i}$ is the volume fraction of polymer *i* and $\chi_{12}$ the interaction parameter. In addition, certain polymer blend combinations show a lower critical solution temperature (LCST) or upper critical solution temperature (UCST) behavior depending on the temperature at which miscibility changes from miscible to immiscible. Furthermore, even if the thermodynamic conditions are favorable, other factors, such as viscosity, may also affect miscibility. Therefore, complete miscibility of the polymers is not easily gained, and they tend to phase separate [52]. 

Phase separation is normally evidenced by a distinct interface between the phases and can be manifested by the glass transition temperature, T_g_, in which a miscible blend shows a single T_g_ whereas an immiscible blend typically shows two T_g_s. The gas separation properties of immiscible blends can be strategically tailored by varying the polymer combination, component compositions, and by controlling the membrane microstructure. Unlike the miscible blends, the phase-separated nature of immiscible blends requires the addition of compatibilizers to further tune the blend morphology and to enhance gas separation properties. Herein, it is important to review immiscible blend membranes with and without compatibilizers and how the blend morphology correlates with the gas separation properties.

Compared to the miscible blend membranes, immiscible blend membranes offer the potential to further enhance gas permeability. Kim et al. [70] studied the O_2_/N_2_ gas permeation and thermodynamic properties for blends of polycarbonate (PC) and polymethylmethacrylate (PMMA). They found that by controlling the annealing temperature and time, the miscibility of the blend could be manipulated. Blend membranes with different morphologies were prepared using the same chemical components and composition, and their N_2_ and O_2_ permeation properties were examined at 35 °C and 1 atm. The results show that the gas permeability and diffusion coefficients decreased in the following order: Immiscible blend having a domain–matrix structure > immiscible blend having an interconnected structure > miscible blend. It is suggested that these results may be related to differences in local polymer chain motions that depend on the level of intermolecular mixing.

**Figure 4:**
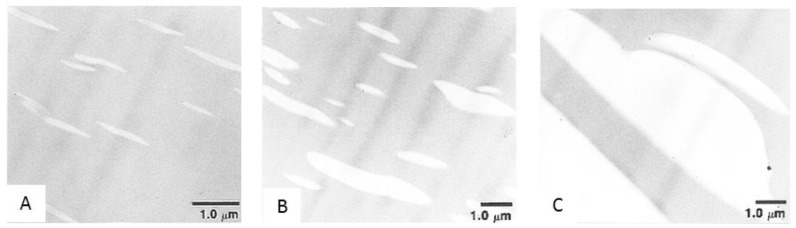
Transmission electron micrographs of PTMSP/PPP blends at three blend compositions (in wt % PPP):  (**A**) 3; (**B**) 10; (**C**) 25. The PTMSP-rich phase is electron-opaque and dark, and the PPP phase is bright. (Adapted with permission from Panapitiya, T.L.G.; Freeman, B.D.; Spontak, R.J.; Morisato, A.; Pinnau, I. Gas Permeability and Phase Morphology of Poly(1-(trimethylsilyl)-1-propyne)/Poly(1-phenyl-1-propyne) Blends. *Macromolecules* **1997**, *30*, 4766–4769. Copyright 1997 American Chemical Society.)

The gas transport properties are strongly correlated to the blend morphology for immiscible blend membranes. Toy et al. [71] prepared blend membranes consisting of poly(1-(trimethylsilyl)-1-propyne) (PTMSP) and poly(1-phenyl-1-propyne) (PPP) for CO_2_/N_2_ separation. PPP, as the dispersed phase, is less permeable, but more selective, compared to PTMSP. Therefore, increasing the PPP concentration in the blend leads to significant decreases in CO_2_ and N_2_ permeability and a dramatic increase in the CO_2_/N_2_ selectivity. As illustrated in Figure 4, when the PPP concentration was increased from 3 wt % to 25 wt %, the dispersed ellipsoidal domains expanded and started to coalesce to form effective barriers for gas diffusion, which accounts for the decrease in gas permeability and the increase in CO_2_/N_2_ selectivity.

Li et al. [72] fabricated blend membranes of poly(4-vinylpyridine) (PVP) and ethylcellulose (EC) to study gas transport properties for O_2_, N_2_, CO_2_, CH_4_, and H_2_. The blend membranes were macroscopically miscible, but microscopically immiscible, with PVP as the dispersed phase and EC as the continuous phase when the PVP content is less than 50 wt %. With increasing PVP content, the permeability decreased for all of the tested gases and the permselectivities increased due to the increased domain size and number of dispersed PVP phase. For blend membranes with PVP content ranging from 50 to 60 wt %, phase inversion may occur leading to PVP becoming the continuous phase, which accounts for the decreasing permeability and increasing permselectivity. It is claimed that the PVP/EC blend membranes inherited the improved mechanical properties, membrane-forming ability, and high gas permeability from the EC phase, and the high permselectivity from the PVP phase. 

More than varying blend composition, Sales et al. [73] investigated the effects of temperature and pressure on the gas transport properties of polyurethane (PU) and poly(methylmethacrylate) (PMMA) blend membranes. With 30 wt % PMMA in the blend, the permeability and diffusion coefficients of CO_2_, H_2_, O_2_, CH_4_, and N_2_ decrease approximately 55% due to the reduction of free-volume. The effects of temperature on gas permeability and selectivity were found to be significant. Increasing the temperature from 10 to 40 °C results in a 30% increase in free volume that leads to increases in gas permeability and diffusivity, and gas selectivity for all gas pairs can be increased at low temperature. On the other hand, the testing pressure has little effect on gas transport properties, especially in the membranes with high PMMA content. Thus, the gas separation properties of immiscible polymer blends can be tailored by changing component compositions and by controlling the membrane morphology.

## 4. Morphology Control and Compatibilizers for Gas Separation Membranes

**Figure 5:**
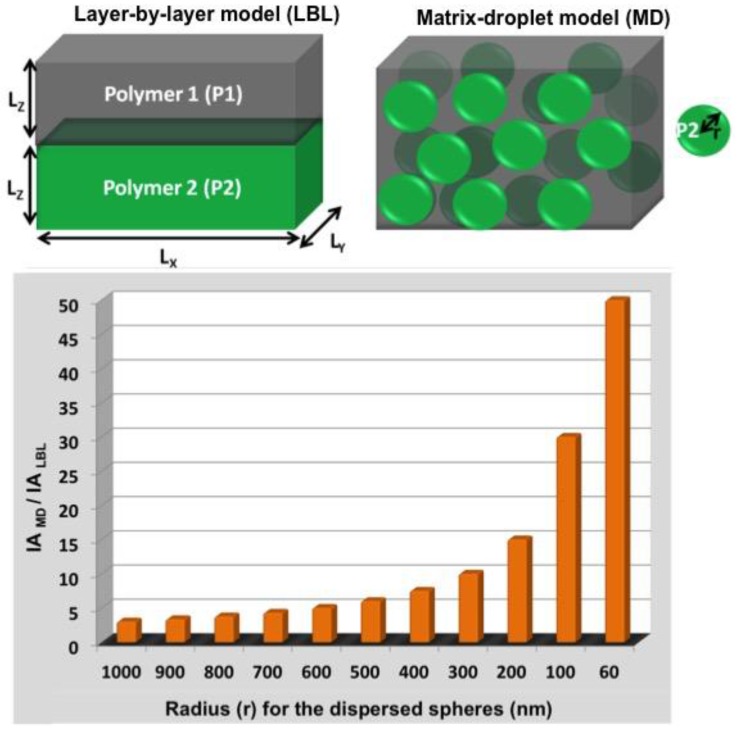
Cartoon showing the LBL and MD morphologies and a histogram of IA_MD_/IA_LBL_ with respect to the radius of the dispersed phase.

One important advantage provided by the immiscible polymer blends compared to miscible blends is that the immiscible blends allow for control of membrane morphology. The matrix-droplet (MD) membrane morphology provides a higher interfacial surface area compared to a layer-by-layer morphology (LBL). This can be explained using the model shown in Figure 5. 

The plot shows the variation in the ratio of the interfacial area (IA) of the MD morphology with respect to the LBL morphology as a function of the domain size of the dispersed phase in the MD morphology (IA_MD_/IA_LBL_ = 3L_Z_/r). For a hypothetical membrane 1 µm thick, one sees that as the size of the dispersed phase becomes smaller, this ratio increases [74]. One of the ways to lower the domain size of the dispersed phase is to use a compatibilizer. It has been demonstrated [52] that, when the compatibilizer concentration increases up to a certain threshold, the domain size can be decreased. This enhancement of the interfacial area is vital for improving permeability in gas separation membranes. However, how does one improve gas pair selectivity? Panapitiya et al. recently reported a novel membrane microstructure in which the dispersed phase is comprised of a highly permeable polymer, while the continuous phase is a highly selective polymer [58]. The argument is that the adsorbed gases passing through the highly permeable, though poorly selective, dispersed phase must subsequently pass through a thin, highly selective continuous phase to traverse the membrane. This transport pathway ensures that both the high selectivity of the continuous polymer matrix and the high permeability of the dispersed phase are utilized. This droplet-matrix morphology was obtained using two high performance polymers, PBI (polybenzimidazole) and 6FDD (6FDA-DAM-DABA, a copolymer of 4,4-hexafluoroisopropylidene diphthalic anhydride (6FDA), 2,4,6-trimethyl-1,3-phenylenediamine (DAM) and 3,5-diaminobenzoic acid (DABA)), since these materials are capable of withstanding industrially relevant conditions of temperature and pressure, e.g., 300 °C and 30 atm, for applications such as H_2_ separation from CO_2_ [74]. The otherwise immiscible polymers were compatibilized using the commercially available MOF, ZIF-8.

**Figure 6:**
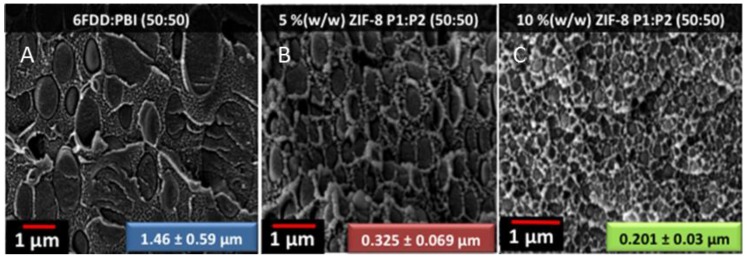
SEM images of cross-sections of a 6FDA-DAM:DABA/PBI (50:50) membrane without ZIF-8 (**A**); with 5 wt % ZIF-8 (**B**); and with 10 wt % ZIF-8 (**C**). (Adapted with permission from Panapitiya, N.P.; Wijenayake, S.N.; Huang, Y.; Bushdiecker, D.; Nguyen, D.; Ratanawanate, C.; Kalaw, G.J.; Gilpin, C.J.; Musselman, I.H.; Balkus, K.J.; Ferraris, J.P. Stabilization of immiscible polymer blends using structure directing metal organic frameworks (MOFs). *Polymer* **2014,** *55*, 2028–2034. Copyright 2014 Elsevier.)

**Figure 7:**
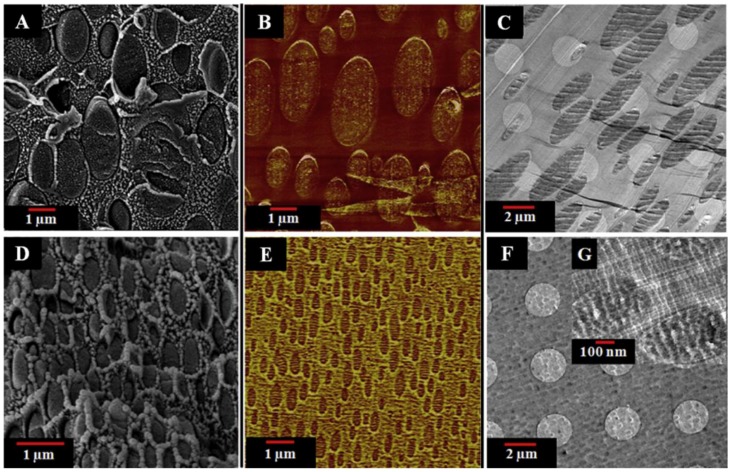
(**A**) SEM, (**B**) AFM, and (**C**) TEM images of 6FDD:PBI (50:50) blend membrane cross-sections, and (**D**) SEM, (**E**) AFM, and (**F,G**) TEM images 6FDD:PBI (50:50) containing 5 wt % ZIF-8. (Reprinted with permission from Panapitiya, N.P.; Wijenayake, S.N.; Huang, Y.; Bushdiecker, D.; Nguyen, D.; Ratanawanate, C.; Kalaw, G.J.; Gilpin, C.J.; Musselman, I.H.; Balkus, K.J.; Ferraris, J.P. Stabilization of immiscible polymer blends using structure directing metal organic frameworks (MOFs). *Polymer* **2014,** *55*, 2028–2034. Copyright 2014 Elsevier.)

The SEM images in Figure 6 show that as the ZIF-8 loading is increased from 0 wt % to 10 wt %, the average dispersed domain radius is decreased from 1.46 to 0.201 μm, and the size distribution is decreased as well. This result shows that ZIF-8 works as a compatibilizer. Multiple imaging techniques (SEM, AFM, TEM) were used to view the membrane cross-sections to study the compatibilization afforded by ZIF-8 (Figure 7). All images revealed that the interface is free from non-selective voids, which would lower gas selectivities. 

In order to determine the distribution of ZIF-8 in the membrane the authors used a thermodynamic calculation with the aid of water contact angle measurements. Water contact angles for each component of the compatibilized blend were measured separately in order to calculate the interfacial energies between each polymer and the ZIF-8 particles, and between the polymers. The localization of the particles at the blend’s interface could be surmised on a thermodynamic basis [75,76,77,78]. The wetting parameter (ω) is defined by Young’s equation as:

(9) $$\omega =\cos\theta =\frac{\gamma_{ZIF-8/6FDD}-\gamma_{ZIF-8/PBI}}{\gamma_{PBI/6FDD}}$$

where θ is the contact angle of ZIF-8 at the interface and $\gamma_{ZIF-8/6FDD}$, $\gamma_{ZIF-8/PBI}$ and $\gamma_{PBI/6FDD}$ represent the interfacial tension. When $\left|\gamma_{6FDD/ZIF-8}-\gamma_{PBI/ZIF-8}\right|$ < $\gamma_{PBI/6FDD\;}$, the ZIF-8 would stay mainly at the interface (−1 < ω<1 with 0° < θ<180°). Otherwise, ZIF-8 would preferentially go into either 6FDD or PBI phase when $\left|\gamma_{6FDD/ZIF-8}-\gamma_{PBI/ZIF-8}\right|$ > $\gamma_{PBI/6FDD}$ (ω < −1 or ω >1)

The interfacial tension is derived from Girifalco-Good equation as:

(10) $$\gamma_{PBI/6FDD}=\gamma_{iPBI}+\gamma_{i6FDD}-\sqrt{\left(\gamma_{iPBI}\gamma_{i6FDD}\right)}$$

where $\gamma_{i}$ is the surface tension of the individual constituent as calculated by measuring the water contact angles (WCA) of each constituent, which is defined by the equation below:

(11) $$y_{iS}=\frac{\gamma_{iL}{\left(1+\cos\theta \right)}^{2}}{4}$$

where *L* represents the liquid while *S* the solid and θ is the water-contact angle.

The wettability ω was calculated to be 0.063, which lies in between −1 < ω<1. Hence ZIF-8 particles were predicted to stay at the interface of the immiscible blend polymer components and stabilize the phase separation. These results also were consistent with the decreasing size of the dispersed phase domains observed in the SEM images. ZIF-8 has shown itself as an excellent compatibilizer for this immiscible blend comprising high performance polymers 6FDD and PBI, which are being used widely in membrane research for gas separation.

**Figure 8:**
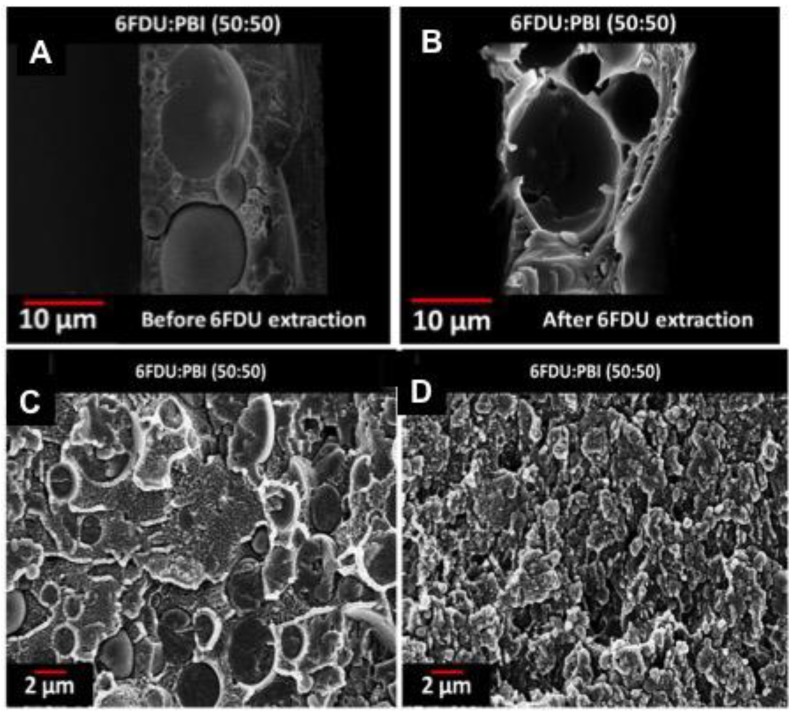
SEM images of the membrane cross-sections of 6FDU:PBI (50:50) blend before (**A**); and after (**B**); selective solvent extraction of 6FDU with 5wt % colloidal ZIF-8 (**C**); and with 17wt% colloidal ZIF-8 (**D**). (Adapted with permission from Panapitiya, N.P. Novel compatibilised immiscible polymer blend based membranes for gas separations. Ph.D. thesis, University of Texas at Dallas, TX, USA, 24 May 2014. Copyright 2014 Nimanka Pathum Panapitiya.)

Panapitiya et al. have extended this approach to another immiscible polymer blend system in which 6FDA-Durene and PBI are compatibilized with as-synthesized colloidal ZIF-8. Once again, upon the addition of c-ZIF-8, the size of the dispersed phase became smaller and more uniform indicating a better compatibility between the polymers (Figure 8) [74].

Other compatibilizers, including co-polymers [53,54,55], nanoparticles [56,57], small molecules [59], and other additives [58], can be used to improve the miscibility of otherwise incompatible polymers and have been the subject of numerous reviews. In this review we focus on the use of the compatibilizers specifically for gas separation applications.

**Figure 9:**
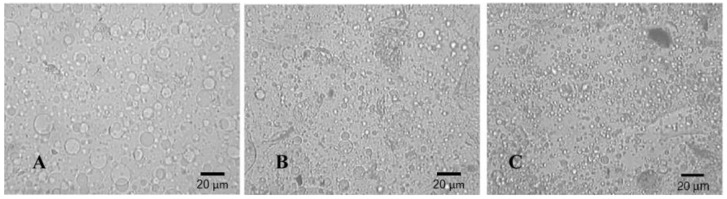
Optical microphotographs of PMMA/PVME=9/1 blends: (**A**) without PS-b-PMMA; (**B**) with 2 phr PS-b-PMMA; (**C**) with 5 phr PS-b-PMMA [79]. (Reprinted with permission from Moon, E.J.; Yoo, J.E.; Choi, H.W.; Kim, C.K. Gas transport and thermodynamic properties of PMMA/PVME blends containing PS-b-PMMA as a compatibilizer. *J. Memb. Sci.* **2002**, *204*, 283–294. Copyright 2002 Elsevier.)

The most used compatibilizers for gas separation applications comprise block copolymers containing segments of one or more of the homopolymers in the blend. Moon et al. [79] studied the gas transport properties of polymethylmethacrylate (PMMA) and polyvinylmethylether (PVME) blend membranes containing the diblock copolymer of styrene and methylmethacrylate (PS-b-PMMA). As seen in Figure 9, the average diameter of the dispersed domains in the blend decreased with increasing compatibilizer concentration up to five parts per hundred (phr) and then leveled off at a fixed value. Similarly, the O_2_ and N_2_ permeabilities increased with increasing compatibilizer concentration up to 5 phr and then leveled off. The size reduction of the dispersed domains gives rise to an increase in the number of the domains and the formation of continuous paths from the top to the bottom of the membrane for the same blend composition, which contributes to the increase in gas permeability. 

**Figure 10:**
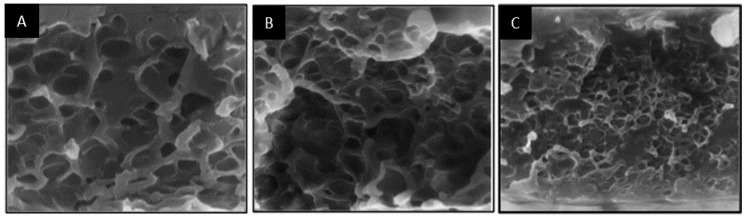
SEM images of the cross-section of PPO/PI (9/1 by wt %) blend membranes: (**A**) 0 wt %; (**B**) 2 wt %; and (**C**) 4 wt % of the SIS block copolymer (2000 ×) [80]. (Adapted with permission from Park, C. Morphological effect of dispersed phase on gas permeation properties through heterophase polymer membrane: Theoretical and experimental approaches. *Polymer* **2000**, *41*, 1765–1771. Copyright 2000 Oxford: Elsevier Science.)

**Figure 11:**
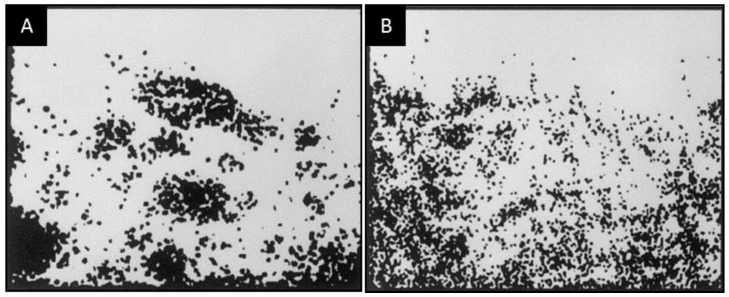
Energy-dispersive X-ray micrographs of the cross-section of PPO/PI (9/1 by wt %) blend membranes: (**A**) 0 wt %; and (**B**) 4 wt % of the SIS block copolymer [80]. (Adapted with permission from Park, C. Morphological effect of dispersed phase on gas permeation properties through heterophase polymer membrane: Theoretical and experimental approaches. *Polymer* **2000**, *41*, 1765–1771. Copyright 2000 Oxford: Elsevier Science.)

Triblock copolymers have also been used as compatibilizers. Park et al. [80] used a styrene-isoprene-styrene triblock copolymer to compatibilize poly(phenylene oxide)/polyisoprene blend membranes for O_2_/N_2_ separation, and Semsarzadeh et al. [81] used polyethylene oxide-polypropylene oxide-polyethylene oxide triblock copolymers to compatibilize polyurethane/polyvinyl acetate blend membranes for CO_2_/CH_4_ and CO_2_/N_2_ separations. Both studies observed similar trends in that the size of the dispersed domains decreases and the gas permeability increases upon increasing the compatibilizer content at the same blend composition. Using SEM and energy-dispersive X-ray spectroscopy, Park et al. [80] also observed that, as the dispersed phase became smaller with increasing concentration of the compatibilizer, the probability of forming continuous paths of the dispersed phase in the blend also increased (Figure 10 and Figure 11).

Metal salts have also been used as compatibilizers. Lai et al. [82] added CuCl_2_·2H_2_O into poly(methy-1-methacrylate) (PMMA)/polycarbonate (PC) blend membranes and observed a change in blend morphology in which the size of the dispersed domain was reduced. Moreover, the gas permeabilities of the PMMA/PC-rich and PMMA-rich/PC membranes with CuCl_2_·2H_2_O are higher than those without CuCl_2_·2H_2_O. These phenomena may be due to the fact that complex formation in the PMMA/PC blend membrane occurs with the addition of salt.

**Figure 12:**
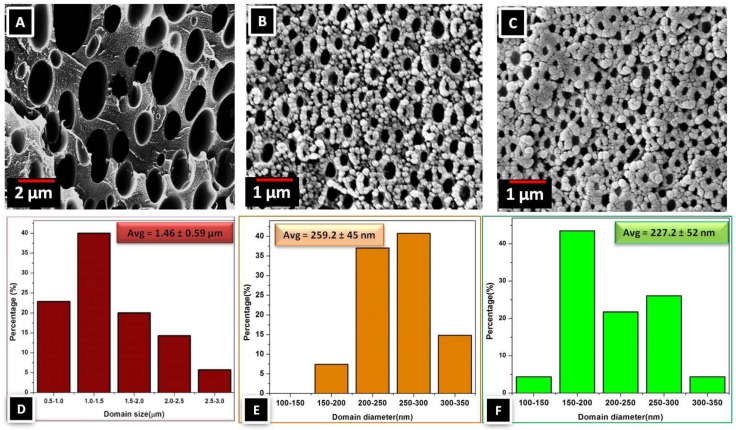
SEM images (**A−C**) and histograms (**D−F**) of the 6FDD-extracted 6FDD:PBI (50:50) membranes containing (**A,D**) 0 wt %, (**B,E**) 5 wt %, and (**C,F**) 9 wt % 2-MI. (Reprinted with permission from Panapitiya, Panapitiya, N.P.; Wijenayake, S.N.; Nguyen, D.D.; Huang, Y.; Musselman, I.H.; Balkus, K.J.; Ferraris, J.P. Gas Separation Membranes Derived from High-Performance Immiscible Polymer Blends Compatibilized with Small Molecules. *ACS Appl. Mater. Interfaces* **2015**, *7*, 18618–18627. Copyright 2015 American Chemical Society Elsevier.)

Recently, for the first time Panapitiya et al. were able to compatibilize the same 6FDD/PBI blend system using small molecules, such as 2-methylimidazole (2-MI) (Figure 12) [59].

**Figure 13:**
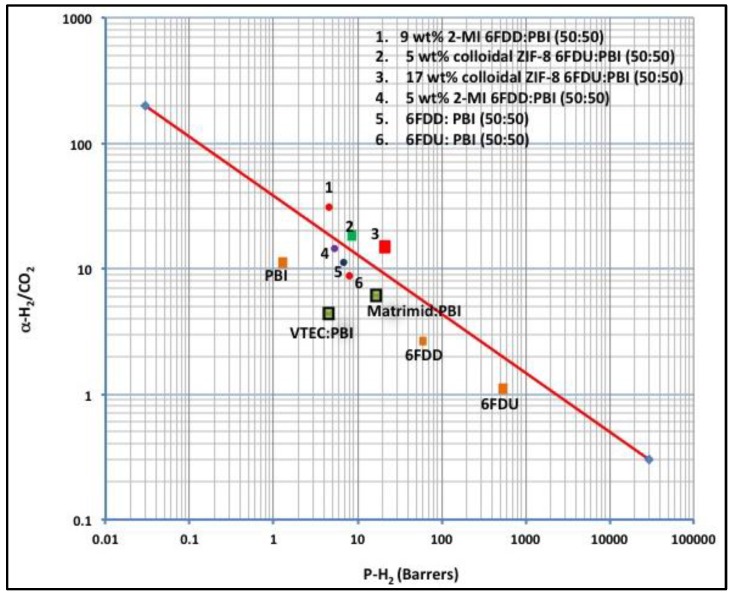
Robeson plot of gas permeability properties of compatibilized immiscible polymer blends for H_2_ separation compared with the several miscible polymer blends.

The compatibilized blend membranes exhibit a more uniform morphology. The incorporation of 2-MI in the blend membrane enhanced the H_2_/CO_2_ selectivity significantly, locating the compatibilized blend membrane above the Robeson upper bound (Figure 13).

These reports show that compatibilizers can stabilize the morphology of immiscible polymer blend membranes, resulting in improved gas permeability properties. This finding opens up the possibility of using many more blend compositions for gas separation membranes.

## 5. Compatibilizers and Improvement in Mechanical Properties (Tensile Strength, Young’s Modulus, Elongation at Break, and Ductility)

In this section of the review, the effect of different types of compatibilizers, including inorganic nanofillers, block co-polymers, and small organic molecules, on immiscible polymer blends will be addressed with a focus on the resulting mechanical properties. In their recent perspective, Baker and Low [83] pointed out the urgent need for new materials to satisfy the actual industrial requirements for gas separation membranes. These materials should be able to be scaled up to areas as large as 500,000 m^2^, while keeping the selective layer thin (e.g., 0.1–1.0 µm), so that both high flux and high selectivity can be achieved [83]. Gas separation usually takes place at a high pressure of ~30 bar and a high temperature above 150 °C [84,85]. Therefore, membrane materials need to possess excellent mechanical properties in order to perform under such extreme conditions. Blending of immiscible polymers has proven its potential for combining the desired properties of each individual polymeric component into a same material platform. However, due to the unfavorable thermodynamics of mixing, immiscible polymer blends tend to macroscopically phase-separate. The resultant blends therefore usually display poor mechanical properties in terms of toughness, flexibility, and ductility. Hence, it is necessary to compatibilize the phase-separating immiscible polymer blends. In this section of the review, the effect of different types of compatibilizers including inorganic nanofillers, block co-polymers and small organic molecules on immiscible polymer blends will be addressed with a focus on the improvements in mechanical properties. Ray et al. reported that the incorporation of 6 wt % of the organoclay C20A (cloisite) led to an increase in modulus with respect to pure blends [86]. The same tensile strengths were observed between compatibilized and non-compatiblized blend membranes but elongation at breaks increased significantly for blend membranes with C20A. Specifically, the pure blend PC/PMMA (40/60) exhibited a Young’s modulus value of 850 MPa whereas the same compatibilized blend with 6 wt % of the nanoclay C20A PC/PMMA(37/57) showed a significant increase in Young’s Modulus ~1550 MPa. The elongation at break of the compatibilized blend also went up from 8.5 to 14 mm. Both PC and PMMA are glassy polymers that have potential for gas separations [70,87,88,89,90]. Further work done by Wang et al. also showed an improvement in mechanical properties of the 70/30 immiscible blend polypropylene/polystyrene (PP/PS) when using an organically modified layered clay (OMMT) as a compatibilizer. Compared to the pure blend, the compatibilized blend with 5 wt % of OMMT showed a much higher peel strength (1768 N/m vs. 523 N/m) as well as larger adhesive fracture energy (3356 J/m^2^ vs. 1046 J/m^2^). In this case study, peel strength was defined as average load per unit width of bond line required to increasingly detach two bonded, flexible materials. Adhesive fracture energy (also known as critical strain energy release rate) was quantified as the amount of energy required to induce crack of materials. The observed behaviors were attributed to the fact that both the polymers PP and PS were inserted in between layers of OMMT, leading to the confining and decrease in the size of PS dispersed phase from ~4 µm to ~0.5 µm [91]. 

Zeolites are known as excellent nanoporous materials for gas separation membranes, exhibiting size and shape selectivity. In addition, zeolites could serve as potential compatibilizers for immiscible polymer blends as well as shown by Thipmanee and co-workers. An immiscible blend of polyethylene/thermoplastic starch PE/TPS (70/30) was compatibilized by zeolite 5 (ZSM5). To fabricate the films, ZSM5 was first introduced into TPS followed by addition of PE to the mixture of TPS/ZSM5. The compatibilized blend with 5wt % of ZSM5 displayed improvement in tensile strength (23.8 MPa vs. 20.6 MPa), Young’s Modulus (600 MPa vs. 158 MPa), elongation at break (407% vs 24.5%) and impact strength (2.1 kJ/m vs. 1.74 kJ/m) when compared to the pure blend. The authors rationalized the overall enhancement in mechanical properties based on the improved dispersion of TPS phase, as well as the ability of ZSM5 to reinforce the micro-mechanical properties of the blend. However, the authors also pointed out that further increased loadings of ZSM5 above 5 wt % ended up reducing the tensile strength of the compatibilized blend due to the aggregation of ZSM5 particles at the interface [92]. 

Pursuing another approach, Cao and co-workers investigated the compatibilizing effects of graphene oxide sheets (GOs) on the immiscible blend of polyamide (PA) and polyphenylene oxide (PPO) with an overall improvement in the ductility of the PA/PPO (90/10) blend [50]. An increase in elongation at break was in accordance with observed yield behavior. When 1 wt % of GOs was added, the elongation at break went up by 89% from 28.6% (PA/PPO) to 54% while the tensile strength of the compatibilized blend increased by 87% from 17.5 MPa to 32.7 MPa. Cao and co-workers attributed this increasing trend in mechanical properties of their blend to the very high Young’s modulus of GOs. The improved mechanical properties of this PA/PPO blend compatibilized with GOs gives them potential for gas separations at high temperature and high pressure as the component polymers have been used for gas separation and the graphene oxide sheets have also served as interfacial reinforcing inorganic fillers [50]. This study was further supported by the work done by Yang et al. [93], in which immiscible blends of nylon 6 and poly(vinylidene fluoride) were compatibilized using graphene oxide (GO). The composition of the blends was kept constant with N6/PVF (90/10) while increasing the loadings of GO from 0.5 to 1 wt % then 2 wt %. As a result of the compatibilizing effect of GO, the elongation at break significantly increased by 200% when the amounts of GO changed from 0.5 to 1 wt %. The improved ductility and strength arose from more uniform and smaller dispersed phase domains [5]. Hence, GO proved to be a potential compatibilizer for immiscible polymer blends. This approach could be attractive for gas separation purposes. In another independent study carried out by Huang and co-workers [94], graphene oxide (GO) was employed to compatibilize the 50/50 immiscible blend of polyamide 6 (PA6) and poly (acrylonitrile-butadiene-styrene) (ABS). The authors observed an increasing trend for both Young’s Modulus and elongation at break as a result of compatibilizing effect of GO. Specifically, the pure blend PA6/ABS had a low Young’ Modulus of 2100 MPa, which went up to 2300 MPa in the same blend with 0.5 wt % GO added. A huge increase in elongation at break was also observed in the 0.5 wt % GO-PA6/ABS blend from 8% of the pure blend to 47%. Furthermore, impact strength also went up from 9 kJ/m^2^ for the pure blend to 22.5 kJ/m^2^ for the blend compatibilized with 0.5 wt % GO. The achieved improvement in mechanical properties of the compatibilized PA6/ABS blend was due to the well-known excellent toughness of GO. However, tensile strength appeared to decrease with increased loadings of GO owing to the agglomeration of GO particles followed by the rigidification of the interface [94]. Another form of carbon-based material was used as a compatibilizer for immiscible polymer blend as reported by Zhao et al. [95] In their work, 80/20 blend of polylactic acid (PLA) and poly(*ε*-caprolactone) (PCL) was compatibilized by addition of multiwall carbon nanotubes (MWCNTs). The resulting compatibilized blend of PLA/PCL with 2 wt % of MWCNTs exhibited an increase in tensile strength (2.82 MPa) and Young’s modulus (125.1 MPa) as compared to the values of the same parameters of the pure blend, which were 2.45 MPa and 81.7 MPa, respectively. A slight decrease in ductility was observed through a small drop in elongation at break of the compatibilized blend (77.6%) when compared to that of the pure blend (96%). This phenomenon could be attributed the fact that MWCNTs tended to aggregate within the blend matrix due to poor dispersion in casting solvents. This problem needs to be further investigated and overcome by the use of functionalized CNTs with additional functional groups, such as carboxylic acids on the surface of the CNT’s walls to facilitate favorable chemical bonding/interactions (i.e., H-bonding). The authors argued that the presence of MWCNTs in the blend matrix not only improved the phase adhesion between PLA and PCL but also facilitated the transfer of stress between the phases. As a result, the concentration of stress was decreased that further increased the tensile properties of the compatibilized immiscible blend [7]. 

Besides inorganic fillers, block copolymers have been intensively studied for compatibilization of immiscible blends. Lee et al. [96] reported that the 70/30 ratio immiscible blend of polycarbonate (PC) and polylactic acid (PLA) was successfully compatibilized by poly(styrene-g-acrylonitrile)-maleic anhydride (SAN) when the loading of SAN was 5 phr (part per hundred resins by weight). The investigators observed an increasing trend for both flexural and tensile strength when the loadings of SAN were slowly increased from 0 to 5 phr. At 5 phr of SAN, the compatibilized blend PC/PLA/SAN displayed a flexural strength of 113 MPa while that of the pure blend PC/PLA was only 100MPa. The tensile strength of PC/PLA/SAN blend was found to be 65 MPa as compared to 53 MPa of the pure blend PC/PLA. In addition, the resultant PC/PLA/SAN exhibited small and uniform domains of the dispersed phase hence this method can be beneficial for gas separation applications [96,97]. In another work led by Castillo-Castro and co-workers [98], low density polyethylene (LDPE) was blended with polyaniline doped with dodecylbenzene sulfonate (PANIDBSA) with the ratio of PANIDBSA being kept constant at 30 wt % while varying the composition of LDPE. The resultant immiscible blend was compatibilized using the co-polymer PE-g-MA (polyethylene-graft-maleic anhydride). As compared to the pure blend LDPE/PANIDBSA (70/30), the compatibilized blend LDPE/PANIDBSA/5 wt % PE-g-MA displayed a large increase in ductility of ~243% from 9.8% to 33.7% [18]. Similarly, Barrami el al. [99] reported the use of poly(styrene-block-polybutadiene-block-poly (methyl-methacrylate)) (SBM) triblock terpolymer as compatibilizer for the immiscible blend of poly(2,6-dimethyl-1,2-phenylene ether) and poly(styrene-co-acrylonitrile) (PPE/SAN) with a variety of blend compositions including 50/50, 60/40, 70/30, respectively. The 60/40 ratio of blend composition was found to possess the optimum viscosity during processing to afford a morphology with low packing density for the PPE domains. For the pure blend PPE/SAN (60/40), elongated PPE domains were observed in SEM images that were further pulled out from the blend matrix due to the unfavorable thermodynamics of mixing. However, upon addition of SBM, smaller domains of PPE were achieved. The authors argued that this phenomenon required larger fracture energy to deform the blend, consequently, increasing the toughness of the compatibilized blend. Critical stress intensity factor, K_IC_, was introduced as a measure for blend’s toughness since beyond the point of K_IC_, the material would crack spontaneously. The pure blend PPE/SAN had a K_IC_ of 1.5 MPa(m)^1/2^ whereas that of the compatibilized blend with SBM was higher ~1.75 MPa(m)^1/2^. This could be due to the fact that SBM surrounded and covered PPE while bridging the PPE and SAN together, which facilitated the transfer of load from PPE to SAN and vice versa. Hence, an increase in critical stress yield was obtained, leading to improved toughness [99].

Recently, Cao et al. [100] introduced the use of a hybrid between copolymer and graphene oxide sheets. Polypropylene-graft-graphene oxide sheets (PP-g-GOs) was synthesized and used as a compatibilizer for the (50/50) immiscible blends of polypropylene (PP) and poly-phenylene oxide (PPO). The resulting compatibilized blends possessed higher elongation and yield strength when the loading of PP-g-GOs was increased (0.5–1.5 wt %). The pure blend PP/PPO had a tensile strength value of 25 MPa, which was increased further to 30 MPa and 36 MPa when the loadings of PP-g-GOs were 0.5 wt % and 1.5 wt %, respectively. An enhanced elongation at break from 32% of PP/PPO to 55% of PP/PPO/1.5% PP-g-GOs was observed, and as a result, enhanced ductility was achieved, which was attributed to the decrease in domain size of the dispersed phase [100]. A similar phenomenon was also reported by Kar and co-workers [101] with the 90/10 immiscible blends of polyvinylidene fluoride (PVDF) and poly (acrylonitrile-butadiene-styrene) (ABS) by using poly(methyl-methacrylate) (PMMA), as well as PMMA-grafted graphene oxide block polymer (PMMA-g-GO) as compatibilizers. The pure blend PVDF/ABS had a tensile strength of 29 MPa with the Young’s modulus ~1216 MPa and a very low elongation at break of 7%. Yet, those values were significantly increased to 43 MPa, 1385 MPa and 21%, respectively, in the same blend compatibilized with 5 wt % of PMMA. In addition, by incorporating only 2.13 wt % of PMMA-g-GO, the authors were able to achieve even higher values for tensile strength (58 MPa) and Young’s Modulus (2115 MPa). A slight drop in elongation at break from 21% of the PMMA compatibilized blend to 16% of the PMMA-g-GO compatibilized blend. Nevertheless, both compatibilizers still provided better ductility as compared to the moderate 7% of the pure blend [101]. In general, the use of block copolymers as compatibilizers can facilitate a better compatibility between the two polymer phases within an immiscible blend. However, this approach is still limited to a certain pairs of polymers being blended since a compatible block copolymer that is similar to at least one component of the blend is required. When taken into account the lengthy and costly process of preparing novel block copolymers, this approach becomes less favorable. 

More recently, in work done by Panapitiya et al., the small organic molecule, 2-methylimidazole (2-MI), was used to compatibilize the phase separation of an immiscible blend of 6FDA-DAM-DABA (3:2) (6FDD)polyimide and polybenzimidazole (PBI) [59]. These two glassy polymers have been intensively studied due to their high permeability (6FDD) and high selectivity (PBI) for H_2_/CO_2_. Without 2-MI, the two polymers macroscopically phase separated. Yet, when the loadings of 2-MI were increased from 5 wt % and then to 9 wt %, the domain size of the dispersed phase (6FDD) became much smaller. The improved compatibility of the two polymers within the blends rendered a more favorable effect not only on gas separation properties but also on the mechanical properties of the blends. When compared to pure polymers, the tensile strength and Young’s modulus of the compatibilized blends with 5 wt % and 9 wt % 2-MI were significantly increased. Specifically, the tensile strength and Young’s modulus of 6FDD/PBI blend were 73.6 MPa and 2.7G Pa respectively. Yet, these two values went up to 86.6 MPa and 3.1 GPa when 5 wt % of 2-MI was added. Much higher tensile strength and Young’s modulus of 115.4 MPa and 3.5 GPa respectively were obtained with the addition of 9 wt % of 2-MI [14]. Panapitiya et al.’s work is also supported by Martins and co-workers [102], when they reported the successful incorporation of smaller carbon-backbone chain of carboxylic acids such as myristic acid (C14) and stearic acid (C18). The compatibilized immiscible blends of polypropylene (PP) and thermoplastic starch (TPS) with small amounts of C14 and C18 exhibited an increase of 17% and 25% in tensile strength at break, as well as 180% and 259% increase in elongation at break, respectively. The highest increase of ~54% in impact strength was obtained when C14 was added. Specifically, the pure blend PP/TPS had a tensile strength of 15.5 MPa. Yet, this value was further improved to 18.9 MPa and 19.31 MPa when C14 and C18 were incorporated into the same blend, respectively. A dramatic enhancement in elongation at break was achieved with 263.2% of C14 and 205.5% of C18 compatibilized blends respectively as compared to the 73.4% elongation at break of the pure blend, suggesting better ductility [15]. This approach could be a better and simpler way to compatibilize immiscible polymer blends with small organic molecules that are cost-effective and readily available. Though not all of the above representative immiscible blends have been used for gas separation, the improved compatibility and mechanical properties make them potentially attractive as a new class of membranes for gas separations that possess good microstructures and toughness for the industrial separation processes at high pressure and high temperature.

## 6. Compatibilized Immiscible Polymer Blends and Commercial Gas Separation Membranes 

Pure polymer based membranes are currently being used in the industry to separate gas mixtures. These polymeric materials are comprised of polysulfones, 2-6-dimethylphenylene oxides, aramids, polyimides, polycarbonates and cellulose acetate [103]. However, their gas transport properties have not reached the state of the art and some undergo membrane plasticization [103].

Therefore, the use of polymer blends could be an alternative considering the superior gas permeability properties and plasticization resistance shown by some of the polymer blends. Industrial membranes are used in the form of hollow fibers since they provide a higher surface area and a high gas flux. Therefore, the ability to convert blends in to this form would be one challenge in the field. It is important to note that, hollow fiber membranes derived from polymer blends have been reported in the literature. Following their work on PBI/Matrimid flat membranes, Hosseini et al. successfully fabricated hollow fiber membranes for H_2_/CO_2_ and CO_2_/CH_4_ separations from the same blend [69]. Moreover, Yong et al. fabricated high performance hollow fiber membranes derived from PIM-1/Matrimid blends for CO_2_/CH_4_, O_2_/N_2_ and CO_2_/N_2_ separations [104]. In another study carried out recently by Dai et al., highly solvent resistant poly(fluoropropylmethylsiloxane)/PEI hollow fiber membranes were fabricated resulting in attractive gas permeability properties for the CO_2_/N_2_ gas pair [105]. In addition, technology of hollow fibers derived from polymer blends is patented indicating the commercial interest of these materials [106,107]. To our knowledge no hollow fiber membrane derived from immiscible polymer blends has been reported. However, partially miscible polymers (PIM-1/Matrimid) have been used to form hollow fibers [104]. Since complete miscibility of polymers is rare, it is unlikely that many commercial membranes with completely miscible blends will be found. Herein, use of compatibilized immiscible polymer blends offers an opportunity to investigate a wider variety of polymers to combine favorable properties. It is also important that compatibilized immiscible polymers offer the unique advantage of microstructure control to afford membrane morphologies like matrix-droplet, with a high interfacial area. One of the main challenges in the field is to find suitable compatibilizers, which are capable of forming domains in a nanometer or smaller range. In order to do that smaller compatibilizers are preferred and the recent reports of the use of small molecules as compatibilizers opens up a potentially interesting and a cost effective way to achieve this goal. However, further research is required to transform this technology into commercial membranes.

## 7. Conclusions Future Directions

Blending is a versatile technique that can be used to combine the favorable properties of more than one polymer to improve the gas permeability and selectivity properties of membranes. The advantages are not limited to enhanced gas separation performances, but also extend to improvements in plasticization resistance as well as mechanical and thermal properties. Obtaining completely miscible polymer systems is rare. To overcome this, immiscible polymer blends can be employed with morphologies suitable for gas separations. The use of compatibilizers has been beneficial in morphology control in immiscible polymer blends for gas separations. However, this area should be explored further in order to obtain membranes to be used commercially. Considering the advantage of controlling microstructure, compatibilized immiscible polymer blends can be the next breakthrough in gas separation membranes.

## Figures and Tables

**Table 1:** Gas separation properties of PBI/Matrimid^®^ (50/50 wt %) blend membranes pyrolyzed at 800 °C [69].

Membrane	Permeability (Barrer)			Ideal Selectivity	
	H_2_	CO_2_	CH_4_	H_2_/CO_2_	CO_2_/CH_4_
PBI/Matrimid (50/50 wt %)	112.12	36.60	0.278	8.85	131.65
PBI/Matrimid (50/50 wt %) cross-linked	91.0	3.71	0.104	24.52	30.50
